# Characterization and proteomic profile of extracellular vesicles from peritoneal dialysis efflux

**DOI:** 10.1371/journal.pone.0176987

**Published:** 2017-05-10

**Authors:** Laura Carreras-Planella, Jordi Soler-Majoral, Cristina Rubio-Esteve, Sara Inés Lozano-Ramos, Marcella Franquesa, Josep Bonet, Maria Isabel Troya-Saborido, Francesc Enric Borràs

**Affiliations:** 1 REMAR-IVECAT Group, "Germans Trias i Pujol" Health Science Research Institute, Can Ruti Campus, Badalona, Spain; 2 Department of Cell Biology, Physiology and Immunology, Autonomous University of Barcelona, Barcelona, Spain; 3 Nephrology Department, "Germans Trias i Pujol" University Hospital, Can Ruti Campus, Badalona, Spain; 4 Department of Medicine, Autonomous University of Barcelona, Barcelona, Spain; The University of Tokyo, JAPAN

## Abstract

Peritoneal Dialysis (PD) is considered the best option for a cost-effective mid-term dialysis in patients with Chronic Renal Failure. However, functional failure of the peritoneal membrane (PM) force many patients to stop PD treatment and start haemodialysis. Currently, PM functionality is monitored by the peritoneal equilibration test, a tedious technique that often show changes when the membrane damage is advanced. As in other pathologies, the identification and characterization of extracellular vesicles (EVs) in the peritoneal dialysis efflux (PDE) may represent a non-invasive alternative to identify biomarkers of membrane failure. Using size-exclusion chromatography, we isolated EVs from PDE in a group of patients. Vesicles were characterized by the presence of tetraspanin markers, nanoparticle tracking analysis profile, cryo-electron microscopy and mass spectrometry. Here, we report the isolation and characterization of PDE-EVs. Based on mass spectrometry, we have found a set of well-conserved proteins among patients. Interestingly, the peptide profile also revealed remarkable changes between newly enrolled and longer-treated PD patients. These results are the first step to the identification of PDE-EVs based new markers of PM damage, which could support clinicians in their decision-making in a non-invasive manner.

## Introduction

Peritoneal Dialysis (PD) is a renal replacement technique based on the semipermeable characteristics of the peritoneal membrane (PM). This membrane is composed by a monolayer of mesothelial cells and an interstitial matrix with a high number of capillaries that, in the presence of hyperosmotic PD fluids, permits the removal of small, medium and, to a lesser extent, large molecules, as well as water ultrafiltration. Prolonged exposure to PD fluids, the low pH of the solutions, as well as episodes of peritonitis or haemoperitoneum can cause detachment of mesothelial cells, fibrosis and neovascularization of the PM, resulting in functional degradation. Although the mechanisms of peritoneal fibrosis are still under investigation, one of the most accepted hypotheses is the epithelial to mesenchymal cell transition[1,2], involving factors such as vascular endothelial growth factor (VEGF) or tumour growth factor-β (TGF -β) (reviewed in[3]). Thus, despite PD is considered the best alternative for cost-effective sustainability of dialysis treatment[4,5], different changes ultimately lead to the failure of ultrafiltration of the PM, causing many patients to discontinue their treatment.

Monitoring the PM's functional state is therefore of outstanding importance for patients' management. Currently, PM is monitored based on the 4-hour lasting peritoneal equilibration test (PET). PET data inform about the permeability and transfer characteristics of the PM, estimating the water transport secondary to osmotic changes in the peritoneal cavity. These data allow clinicians to estimate the peritoneal transport, set the dose and type of PD required for each patient, and monitor the function of the PM. However, PET data render a delayed vision of the status of the PM, as the functional failure only occurs in advanced fibrotic lesions. Thus, monitoring early changes may help to identify and prevent functional worsening of the PM, thus helping the clinician to apply the appropriate therapeutic tools to extend their functionality. In this sense, efforts have been made on the proteomic analysis of peritoneal dialysis efflux (PDE)[6–8].

In recent years, the study of extracellular vesicles (EVs) has gained enormous interest in the diagnostic and therapeutic scenarios[9]. EVs are lipid-bilayered vesicles of 50 to 200 nm in diameter produced by most cells, mainly containing proteins, RNAs and metabolites[10]. EVs’ main function is related to cellular communication[10,11], but as their specific composition varies depending on the physiological and functional state of the producing cells, they have been extensively reported as potential biomarkers in a variety of diseases, including those of the renal system[12]. It is conceivable that the cells of the PM respond to the dialysis treatment by secreting EVs, and that these EVs change their composition reflecting the physiological state of the compartment of origin.

Here, our aim was to identify, isolate and characterize PDE-EVs of patients on PD. The results show that PDE is a non-invasive feasible material to isolate EVs using conventional, clinically applicable techniques. Analyses of PDE-EVs content permitted the identification of specific peptide profiles that changed according to time on dialysis. Thus, the study of EVs present in the PDE opens a new line of research to find non-invasive potential biomarkers for the early detection of PM damage in PD patients.

## Materials and methods

### Patients

The Ethical Committee of “Germans Trias i Pujol” Hospital approved the study, and all subjects gave their written consent according to the Declaration of Helsinki[13]. Inclusion criteria were patients over 18 years old diagnosed of a renal disease requiring PD as chronic renal replacement therapy. Patients starting PD due to heart failure, or those showing a peritonitis episode in the previous two months were excluded. Also, patients showing changes in the peritoneal membrane transport type compared to their initial PET or patients showing ultrafiltration failure were also excluded. Nine patients (56% female) from our PD unit were considered for the study. No patients presented any peritonitis episodes in the 2 months previous to the study. Renal diseases included: 2 renal polycystic disease, 2 tubulointersticial nephritis, 4 glomerulonephritis and 1 unknown aetiology. Seven patients were on Continuous Ambulatory Peritoneal Dialysis (CAPD) and 2 patients were on Automated Peritoneal Dialysis (APD). Seven patients were treated with icodextrin. Clinical and laboratory variables, Peritoneal Equilibration Test (PET), type of peritoneal dialysis solution, and total Kt/V as well as peritoneal Kt/V and renal Kt/V were evaluated.

### Peritoneal equilibration test

The Peritoneal Dialysis Unit routinely perform PET monitoring to each patient one month after the begining of the treatment, and then repeat the test approximately every 6 months. In this study, samples used for EV analyses were obtained at the same time that a routine PET was perfomed. All patients included in the study were stable as for the PET functional result (ie, no changes were detected from their initial test). Samples were obtained between June and September 2014.

Peritoneal equilibration tests were performed with 3.87% glucose solution. The test bag was drained and reinfused at 60 min as reported[14]. A blood sample was withdrawn at 240 min and dialysate samples were taken from the pre-infusion bag, and at 0, 60, 120, and 240 min. Urea, creatinine, glucose, sodium, and potassium were analysed in all samples; urate, phosphate, total protein, albumin, were analysed in blood and dialysate samples at 240 min (Cobas 711 Roche diagnostics, Switzerland). A correction was applied for plasma water concentration for small solutes in the blood sample and, in the dialysate sample, creatinine concentration was corrected for the presence of glucose.

### Calculations

Dialysate to plasma (D/P) ratios for urea and creatinine, and dialysate to baseline dialysate ratios (D/Do) for glucose were calculated. The mass transfer area coefficients (MTAC) for urea, creatinine, glucose, urate, phosphate, and potassium were calculated according to Waniewski et al. [15], using F = 0.5. Peritoneal clearances of total protein and albumin were also calculated. All parameters were corrected for 1.73 m^2^ surface area.

Ultrafiltration in PET at 240 min was calculated as the difference between the drained volumes and the initial volume, as follows:

$$Uf_{240\text{ min}}\;\left(\text{ml}\right)=\left(V_{t}\right)-\left(V_{0}\right)$$

where Uf, ultrafiltration; t, time (min); V, volume (mL).

### Statistical analyses of patient data

Data are presented as median (rank). Quantitative data of the two groups were compared using U-Mann-Whitney test, while qualitative data of the groups were analysed using Fisher’s test. (SPSS, version 18.0, Chicago, IL, USA). Statistical significance was defined as *p*<0.05.

### Isolation of EVs from PDE

Isolation of EVs from the concentrated PDE was based on a modification of a previous method described by our group[16]. Five hundred mL PDE were centrifuged at 3,000 *g* for 5 min immediately after collection. The supernatant was filtered through a 0.2 μm filter and concentrated using a Centricon plus-70 filter unit (100 kDa cut-off; Millipore, Bedford, MA). In brief, supernatants were loaded onto the Centricon filter and centrifuged at 2,800 *g* for 30 min. This step was repeated using one filter unit for each sample until the total volume was processed. The retained volume (ranging from 0.8 mL to 2 mL) of concentrated PDE was loaded onto a size-exclusion chromatography (SEC) column.

### Size-exclusion chromatography

Up to 2 mL of concentrated PDE samples were loaded onto 12 mL of Sepharose-CL2B (Sigma-Aldrich, St. Louis, MO, USA) columns equilibrated in citrate buffer (phosphate-buffered saline, PBS/0.32% citrate) and eluted with PBS. Immediately after, up to 20 fractions of approximately 0.5 mL each were collected and keep at -80°C until further use.

### Protein concentration

The protein concentration was measured by Bradford assay (10 μL of sample; Bio-Rad laboratories, USA) with a standard linear curve based on bovine serum albumin (BSA) (Sigma Aldrich).

### Flow cytometry

Flow cytometry was used to identify fractions containing EVs according to their tetraspanin content and performed as reported before[16]. Antibodies anti-CD9 (1:10, Clone VJ1/20), anti-CD63 (1:10, Clone TEA 3/18), or polyclonal isotype (1:5000, Abcam (ab37355), Cambridge, UK) were added to samples an incubated at 4°C for 30 min. After two washes, beads were incubated with FITC-conjugated secondary antibody (SouthernBiotech, Birmingham, AL) and analysed in a FacsVerse flow cytometer (BD Biosciences, San Jose, CA). Approximately 10,000 beads/sample were acquired and analysed using the Flow Jo software (Tree Star, Ashland, OR). In all samples, the top three tetraspanin-containing chromatographic fractions (those containing EVs) were pooled and used in experiments thereafter.

### Sodium dodecyl sulphate-polyacrylamide gel electrophoresis

Protein content profile of EV fractions was determined using sodium dodecyl sulphate-polyacrylamide gel electrophoresis (SDS-PAGE). Ten μL of each sample were diluted in the same volume of Laemmli buffer (2x; Bio-Rad) with β-mercaptoethanol (5%; Bio-Rad) and boiled at 95°C for 10 min. Then, 20 μL of the mix were loaded into a gel (Mini-Protean TGX gel; 10% polyacrylamide; Bio-Rad). Electrophoresis was performed for 1 hour at 150V. Gels were the stained with SilverQuest (Invitrogen) following the manufacturer's instructions.

### Nanoparticle tracking analysis

To determine the concentration and size distribution of EVs, nanoparticle tracking analysis (NTA) was performed in a Nanosight LM10 (Malvern Instruments Ltd, Malvern, UK) with charge-coupled device (CCD) camera (model F-033) and a 638 nm laser. Data were analysed with the NTA V3.0 software. Samples were diluted 10- to 40-fold with 0.2 μm-filtered PBS to yield 40 to 120 particles/frame as recommended by the manufacturer. Up to 3 videos of 60 seconds each were recorded for each sample with the camera shutter at 30.02 ms, gain set at 650 and camera level at 16. Blur and Max Jump Distance were set automatically, detection threshold was set to 5.

### Cryo-electron microscopy

Ten μL of pooled tetraspanin-peak fractions were used for cryoelectron microscopy (cryo-EM). Each sample was laid on Formvar-Carbon EM grids, frozen and immediately analysed with Jeol JEM 2011 transmission electron microscope equipped with a 626 Gatan cryoholder operating at an accelerating voltage of 200 kV. The samples, maintained at -182°C during imaging, were recorded on a Gatan Ultrascan cooled CCD camera under low electron dose conditions to minimize electron bean radiation. The ImageJ software (NIH) was used to measure EVs size.

### Mass spectrometry analysis

Protein content of PDE-EV-enriched fractions was analysed by liquid chromatography followed by mass spectrometry (LC-MS/MS) on a LTQ Orbitrap Velos (Thermo Fisher, Carlsbad, CA). Samples were reduced with DTT, alkylated with ioidoacetamide and precipitated with trichloroacetic acid. The samples were then washed with acetone and reconstituted in urea before an overnight digestion with trypsin.

### Proteomic data processing and analysis

Raw data files were analysed with Max Quant software[17] (version 1.5.3.30) against Uniprot human database (downloaded on December 11, 2015, 70,076 proteins). Parameters set for single protein identification include: (i) minimum peptide length of 7; (ii) maximum false discovery rate (FDR) for peptides and proteins of 1%; (iii) minimum peptides per protein of 1 and minimum unique peptides per protein of 0; (iv) the minimum score for modified peptides was set to 40; (v) main search error of 4 ppm. In addition, in all searches cysteine carbamidomethylation was established as a fixed modification and methionine oxidation and acetylation of the N-terminus were established as variable modifications, with a maximum number of modifications per peptide set to 5. Proteins identified as potential contaminants, those only identified by site or by a reverse sequence, as well as proteins with less than 2 unique peptides were not further considered.

Further analyses of proteins were made using the Intensity-Based Absolute Quantification (iBAQ) values obtained from MaxQuant, and analysed using Perseus software[18] (version 1.5.6.0), InteractiVenn [19] and the EVs specific databases EVpedia [20], Exocarta [21] and Vesiclepedia [22].

iBAQ values were logarithmized to perform the subsequent analysis such as correlation plots, hierarchical clustering analysis (HCA), Principal Component Analysis (PCA) and volcano plot. Gene Ontology (GO) terms for biological process and cellular components were annotated using Perseus. For PCA, data imputation to substitute non-quantified values with low valid intensities based on normal distribution (down-shift of 1.8 and distribution width of 0.3) was performed. Non-supervised HCA was also done after data imputation. Additional HCA was performed considering only the 63 "core" proteins shared by all samples, in both cases after data normalization with z-score and using Euclidean distance in columns and rows. A volcano plot was used to identify the most significant proteins by plotting fold-change difference of log2 iBAQ on x axis and -log2 (p-value) on y axis. The two-sided unpaired t-test was performed with FDR set at 0.05 and s0 at 0.1.

## Results

### Clinical and epidemiological characteristics of dialysis patients

The study included 9 patients divided in two groups depending on the time on PD: patients with less than 10 months on PD (Newly-Enrolled Patients or NEPs), and patients on PD for more than 18 months (Longer-Treated Patients or LTPs). Clinical data are summarized in Table 1 and detailed per patient in S1 Table. Only 1 patient of the LTP group had type 2 diabetes mellitus, while 3 NEPs and 4 LTPs had hypertension. Regarding the modality of PD, CAPD was used in all NEPs and in 3 patients LTPs. Two LTPs patients used APD. No statistical differences were observed in any of these parameters between both groups.

**Table 1:** Basal characteristics of the patients.

n = 9 patients	NEPs <10 months (n = 4 patients)	LTPs >18 months (n = 5 patients)	P-value^b^
**Age (years)**	53.5 (42.0–62.0)^a^	54.0 (27.0–75.0)^a^	0.806
**Time on PD (months)**	7.0 (5.0–10.0)^a^	24.0 (21.0–67.0)^a^	0.0001
**DM (n)**	0	1	1.000
**HTA (n)**	3	4	1.000
**CAPD/APD (n)**	4/0	3/2	0.444
**Icodextrin (n)**	3	4	1.000

^a^Median (rank)

^b^ *p*-values for quantitative data were calculated using U-Mann-Whitney test while qualitative data of the groups were analysed using Fisher’s test.

DM, diabetes mellitus; HTA, hypertension; CAPD, Continuous Ambulatory Peritoneal Dialysis; APD, Automated Peritoneal Dialysis.

Based on PET results (summarized in Table 2 and detailed in S2 Table), 2 NEP patients were classified as "medium transport" and the other 2 NEP patients as "high transport". In the LTP group, 3 patients were classified as "low transport" and 2 patients as "medium transport". The median 4-hour ultrafiltration was 402 (676–82) mL, and the median of total Kt/V was 2.14 (2.37–1.78). Again, no statistically significant differences were found.

**Table 2:** PET characteristics of the patients.

n = 9 patients	NEPs <10 months (n = 4 patients)	LTPs >18 months (n = 5 patients)	P-value ^b^
**D/P creatinine**	0.75 (0.64–0.89) ^a^	0.56 (0.47–0.75) ^a^	0.190
**D/P urea**	0.84 (0.75–0.87) ^a^	0.79 (0.76–0.89) ^a^	1.000
**D/D**_**0**_ **glucose**	0.22 (0.20–0.26) ^a^	0.35 (0.24–0.39) ^a^	0.063
**UF 240 min (mL)**	310.0 (82.0–482.0) ^a^	577.0 (116.0–676.0) ^a^	0.286

^a^ Median (rank)

^b^ *p*-values were calculated using U-Mann-Whitney test

### Isolation of PDE-EVs from PD patients

**Fig 1:**
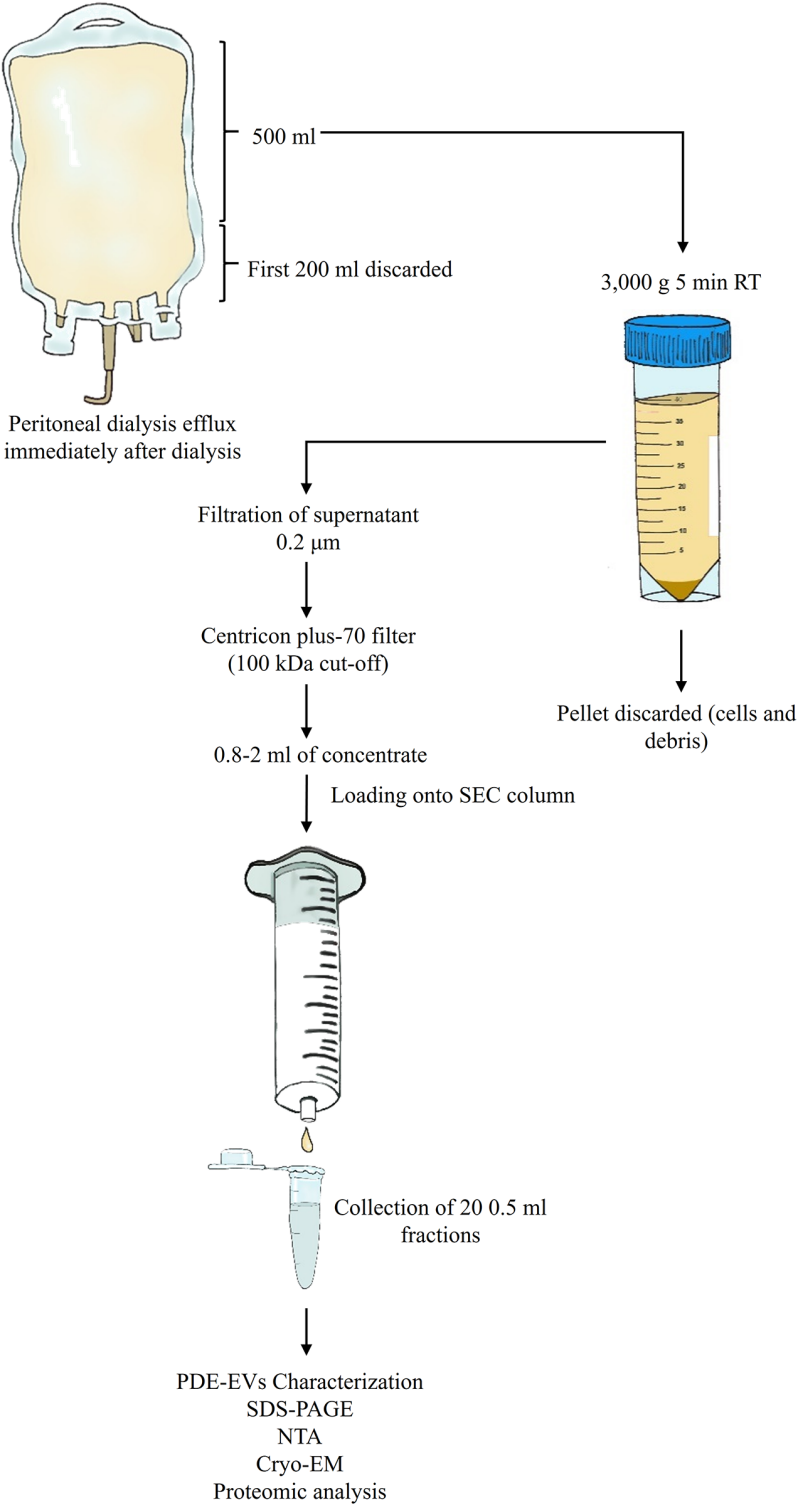
Schematic representation of peritoneal dialysis efflux sample processing and EV isolation.

**Fig 2:**
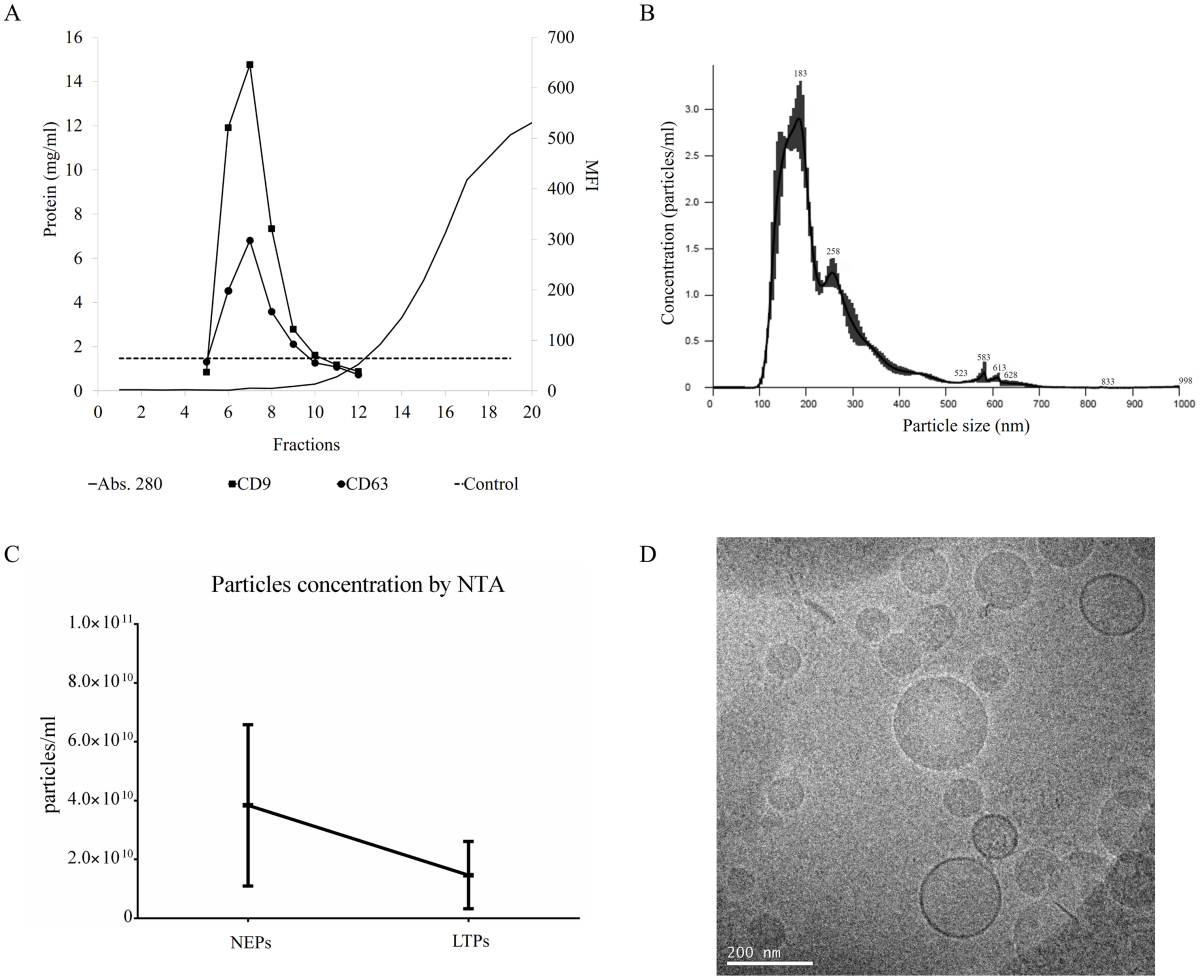
Characterization of PDE-EVs. PDE concentrated samples were further separated using SEC. Up to 20 fractions were recovered and analysed in each sample. In plot A, fractions were analysed for their protein content by BCA (black line). Protein concentration from the different EV-enriched fractions was measured by absorbance at 280nm and calculated using a BSA standard curve. Also, the expression of the EV markers CD9 (black squares) and CD63 (white circles) was determined by flow cytometry. The dotted line represents the isotype control. The left axis represents the total protein content (mg/ml) and the right axis shows the median fluorescence intensity (MFI). For each sample, the three fractions with the highest CD9 and CD63 MFI were pooled for further analyses. A representative plot from 9 experiments is shown. Plot B shows a representative NTA of PDE-EVs (n = 9). Plot C depicts particle concentration determinations, also performed by NTA analyses, in n = 4 NEPs and n = 5 LTPs. Finally, pooled PDE-EVs were visualized by cryo-EM (Fig 2D).

Peritoneal efflux-derived EVs were isolated from patients following a modification of the SEC method (Fig 1). As shown, PDE samples (500 mL) were cleaned from debris, ultra-filtered using a 100 kDa ultrafiltration unit, and loaded into SEC columns. Collected fractions containing higher amount of proteins eluted well-after fraction 10 (Fig 2A). When the same chromatographic fractions were analysed for their tetraspanin markers, CD9 and CD63 expression were found mostly in fractions 6 to 10 (Fig 2A) among the different samples, indicating the presence of PDE-EVs in those fractions.

Then, NTA analyses of these fractions showed that PDE-EVs had a modal distribution mainly ranging from 100 to 200 nm (Fig 2B). Regarding particles' concentration, a faint non-significant reduction in the number of detected particles was found in LTPs compared to NEPs (Fig 2C).

Further confirmation of the presence of PDE-EVs in tetraspanin fractions was obtained using Cryo-EM. Images revealed membrane-limited round shaped vesicles (Fig 2D). All together these data indicated that PDE-EVs could be obtained from NEPs and LTPs undergoing PD.

### Proteomic analysis of PDE-EV fractions

**Fig 3:**
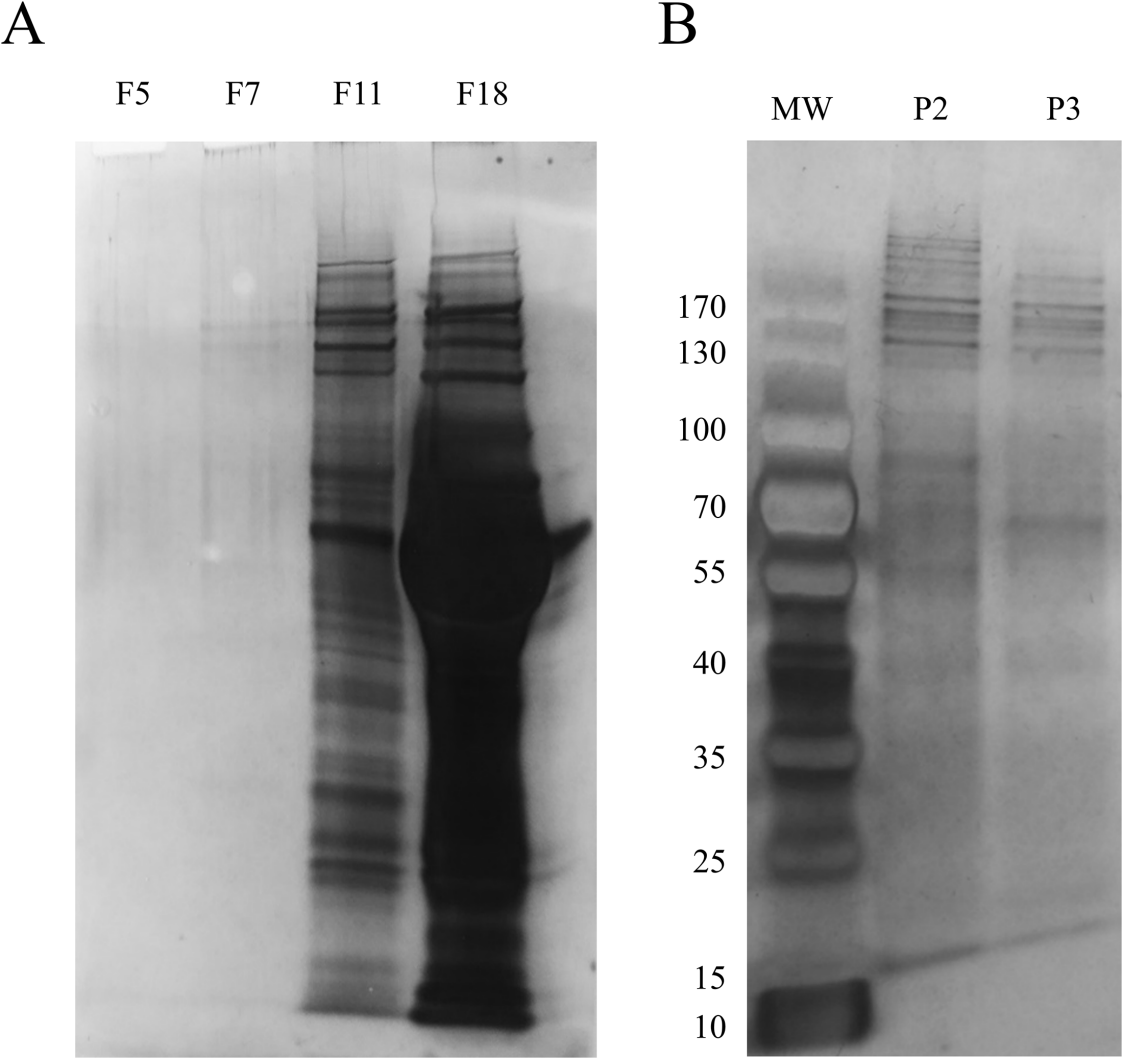
Protein profiling SEC fractions by SDS-PAGE. (A) Silver staining SDS-PAGE of several SEC fractions, including a pre-tetraspanin fraction (F5), a high tetraspanin-containing fraction (F7) and non-EV protein proximal (F11) and distal (F18) fractions. In plot B, pooled tetraspanin-rich fractions from two different experiments (P2 and P3) are shown. Molecular weight markers are also depicted.

As EVs fractions contain low protein amounts, a preliminary protein content analysis was performed using SDS-PAGE experiments to further characterize the PDE-EVs profile. Different fractions from one patient were analysed and silver-stained gels revealed the presence of some bands in fraction 7 (F7, EV-peak fraction), whilst in fractions 11 and 18 (F11, F18, protein fractions) the number and intensity of the bands increased, clearly revealing the presence of bulk proteins (Fig 3A). When pooled EV fractions from each sample (as detected in Fig 2A) were analysed, silver stained gels showed clearer bands although still less abundant compared to protein-containing fractions (Fig 3B, P2 and P3).

**Fig 4:**
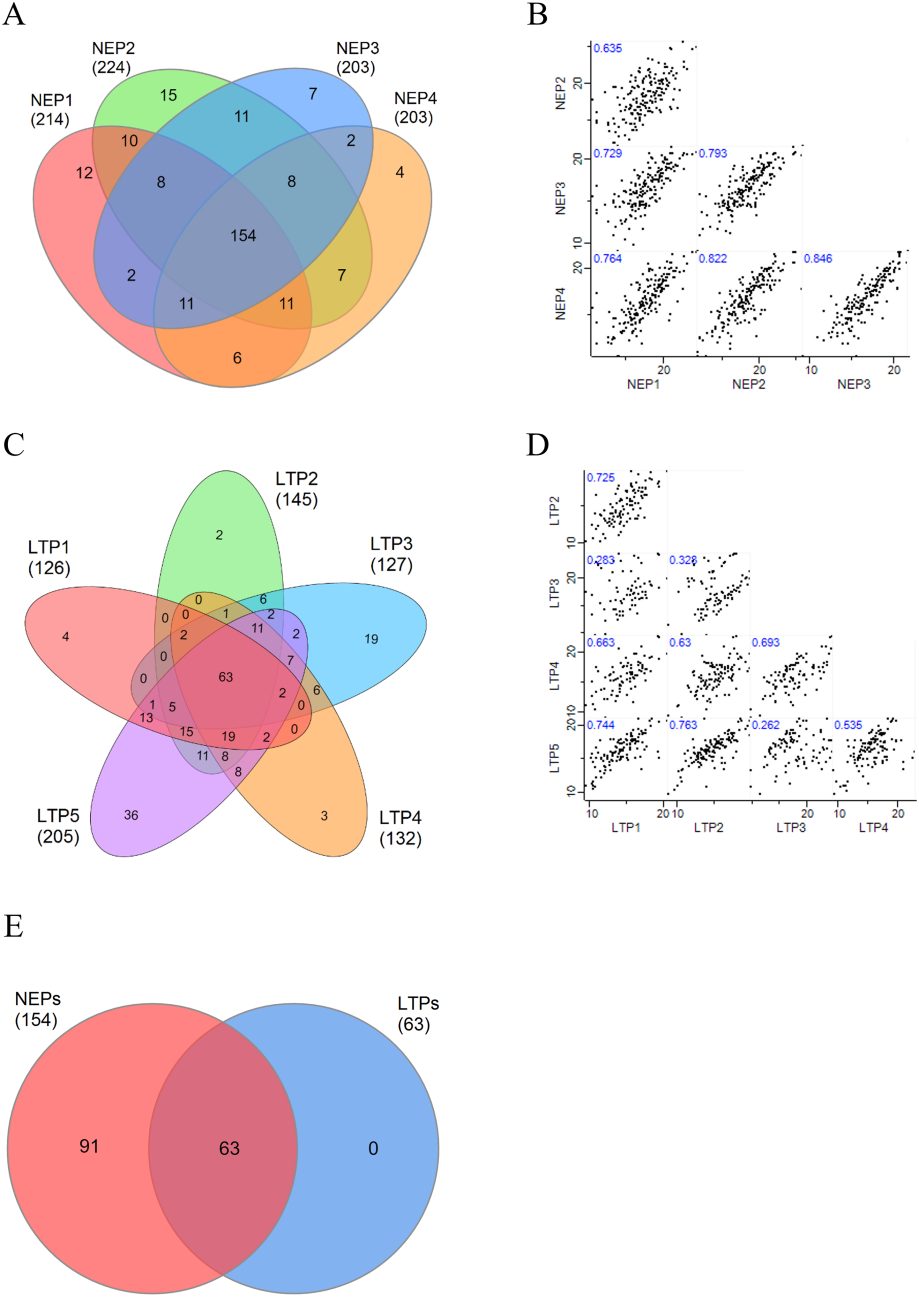
Protein analyses from PDE-EVs. Venn diagrams showing overlapping proteins from n = 4 NEPs (A) and n = 5 LTPs (C) are shown. Correlation multi-scatter plots to analyse the correlation within NEPs (B) and LTPs (D) samples. Pearson Correlation “r” values are labelled on each plot. (E) Venn diagram of the proteins shared by all NEPs compared to the proteins shared by all LTPs.

Peritoneal efflux-derived EVs obtained from each NEP (n = 4) and each LTP (n = 5) were further studied to determine their specific peptide profiles using LC-MS/MS. Only proteins identified by at least 2 unique peptides were considered. Overall, a total of 274 proteins were identified. Among NEPs samples, a mean of 211 proteins were identified (211±8), from which 73% (154 proteins) were found in all patients (Fig 4A), revealing a high intragroup similarity. This was further confirmed by multi-scatter plot showing a Pearson Correlation mean “r” value of 0.76±0.08 (mean±sd) (Fig 4B). Regarding LTPs, a mean of 147 proteins (147±23) were identified, from which only 43% (63 proteins) were shared among all LTPs (Fig 4C), with a Pearson Correlation mean “r” value of 0.56±0.20 (Fig 4D). Interestingly, all 63 proteins shared by LTPs were identified also in all NEPs (Fig 4E). These "core" proteins (listed in Table 3) included proteins unequivocally related to EVs, such as CD81, Galectin 3-binding protein (LGALS3BP), Ezrin (EZR) and several members of the Apolipoprotein (APO) and Annexin (ANXA) families, among others.

**Table 3:** Proteins found in all PDE-EVs samples. Sequence coverage, number of matched peptides, expression fold change between NEPs and LTPs and MS/MS counts are shown for each protein, according to MaxQuant processing of mass-spectrometry data. The proteins are listed in the same order as shown in the clustering analysis in Fig 5. All the proteins present a q-value lower than 10^−3^.

Uniprot entry	Protein name	Gene	Sequence coverage (%)	Matched peptides	Fold Change (NEP/LTP)	Total MS/MS count	MS/MS count								
							NEP1	NEP2	NEP3	NEP4	LTP1	LTP2	LTP3	LTP4	LTP5
P04275	von Willebrand factor;von Willebrand antigen 2	VWF	25.9	60	-1.057	453	1	9	47	70	91	20	122	62	31
P81605	Dermcidin;Survival-promoting peptide;DCD-1	DCD	20.0	2	-0.514	23	1	2	2	1	5	3	2	3	4
P01024	Complement C3;Complement C3 beta chain;C3-beta-c;Complement C3 alpha chain;C3a anaphylatoxin;Acylation stimulating protein;Complement C3b alpha chain;Complement C3c alpha chain fragment 1;Complement C3dg fragment;Complement C3g fragment;Complement C3d fragment;Complement C3f fragment;Complement C3c alpha chain fragment 2	C3	53.9	73	1.536	1426	125	192	175	118	73	263	61	306	113
P02656	Apolipoprotein C-III	APOC3	39.3	3	1.579	69	9	5	4	6	2	1	27	3	12
P08123	Collagen alpha-2(I) chain	COL1A2	15.0	15	2.431	311	49	40	44	34	34	15	20	61	14
P02461	Collagen alpha-1(III) chain	COL3A1	12.6	13	2.734	274	57	39	37	32	16	14	24	44	11
P02452	Collagen alpha-1(I) chain	COL1A1	20.0	23	2.940	343	54	44	54	45	37	8	27	56	18
P02679	Fibrinogen gamma chain	FGG	55.0	26	2.877	1213	219	148	96	137	101	66	190	244	12
P02675	Fibrinogen beta chain;Fibrinopeptide B;Fibrinogen beta chain	FGB	75.6	38	2.804	2020	489	257	116	181	106	68	359	429	15
P02671	Fibrinogen alpha chain;Fibrinopeptide A;Fibrinogen alpha chain	FGA	40.1	28	2.921	639	158	87	50	53	30	27	106	125	3
Q08380	Galectin-3-binding protein	LGALS3BP	37.4	15	3.795	251	20	36	48	47	1	1	67	17	14
P02649	Apolipoprotein E	APOE	61.2	18	3.297	256	22	25	53	40	3	1	72	12	28
P01876	Ig alpha-1 chain C region	IGHA1	53.5	13	2.999	541	48	115	78	52	3	27	132	62	24
P04003	C4b-binding protein alpha chain	C4BPA	57.0	27	3.843	513	59	92	88	52	1	30	150	21	20
B9A064;P0CG04	Immunoglobulin lambda-like polypeptide 5;Ig lambda-1 chain C regions	IGLL5;IGLC1	40.4	7	2.775	262	25	57	27	30	9	14	63	25	12
P01860	Ig gamma-3 chain C region	IGHG3	34.0	12	2.937	198	16	51	23	28	3	5	45	23	4
P01861	Ig gamma-4 chain C region	IGHG4	47.4	10	3.250	45	2	22	2	2	1	1	8	4	3
A0A0B4J1Y9		IGHV3-72	51.5	4	2.773	56	5	13	7	9	3	2	8	5	4
P02647	Apolipoprotein A-I;Proapolipoprotein A-I;Truncated apolipoprotein A-I	APOA1	58.1	16	3.159	257	16	53	42	33	2	1	78	25	7
P98160	Basement membrane-specific heparan sulfate proteoglycan core protein;Endorepellin;LG3 peptide	HSPG2	10.1	32	3.238	111	6	13	26	14	3	41	1	2	5
Q08431	Lactadherin;Lactadherin short form;Medin	MFGE8	47.5	15	2.354	138	16	5	10	37	31	3	2	12	22
P15311	Ezrin	EZR	58.5	34	1.641	607	141	42	45	57	56	126	26	22	92
O00299	Chloride intracellular channel protein 1	CLIC1	66.0	11	1.771	128	29	9	5	16	9	36	5	2	17
O00592	Podocalyxin	PODXL	12.0	7	2.582	57	13	3	7	5	8	4	3	1	13
Q09666	Neuroblast differentiation-associated protein AHNAK	AHNAK	13.8	25	1.953	88	37	3	4	7	12	6	1	1	17
P60903	Protein S100-A10	S100A10	35.1	3	1.981	93	31	6	12	11	8	9	1	2	13
P02751	Fibronectin;Anastellin;Ugl-Y1;Ugl-Y2;Ugl-Y3	FN1	48.8	79	1.726	1930	247	240	260	131	69	486	247	67	183
P06703	Protein S100;Protein S100-A6	S100A6	28.2	3	1.828	63	9	5	8	8	2	13	6	5	7
Q8WUT4	Leucine-rich repeat neuronal protein 4	LRRN4	28.8	16	2.023	368	70	35	34	44	19	66	20	12	68
P68133;P68032;P63267;P62736	Actin, alpha skeletal muscle;Actin, alpha cardiac muscle 1;Actin, gamma-enteric smooth muscle;Actin, aortic smooth muscle	ACTA1;ACTC1;ACTG2;ACTA2	34.0	11	2.057	129	30	12	20	9	4	25	9	8	12
P12110	Collagen alpha-2(VI) chain	COL6A2	17.7	14	2.363	49	6	5	3	7	3	4	1	15	5
P19827	Inter-alpha-trypsin inhibitor heavy chain H1	ITIH1	30.6	18	2.358	461	85	54	62	68	45	40	11	55	41
P19823	Inter-alpha-trypsin inhibitor heavy chain H2	ITIH2	28.3	22	1.711	379	40	44	51	52	33	32	17	46	64
Q53TN4	Cytochrome b reductase 1	CYBRD1	8.7	2	2.267	16	2	2	3	3	1	1	2	1	1
P62987;P62979;P0CG47;P0CG48	Ubiquitin-60S ribosomal protein L40;Ubiquitin;60S ribosomal protein L40;Ubiquitin-40S ribosomal protein S27a;Ubiquitin;40S ribosomal protein S27a;Polyubiquitin-B;Ubiquitin;Polyubiquitin-C;Ubiquitin	UBB;RPS27A;UBC;UBA52;UBBP4	46.2	4	1.752	97	10	12	11	15	10	9	4	10	16
P27487	Dipeptidyl peptidase 4;Dipeptidyl peptidase 4 membrane form;Dipeptidyl peptidase 4 soluble form	DPP4	32.6	25	1.964	272	50	29	13	58	20	30	9	10	53
P00325;P07327;P00326	Alcohol dehydrogenase 1B;Alcohol dehydrogenase 1A;Alcohol dehydrogenase 1C	ADH1B;ADH1A;ADH1C	38.7	12	2.519	92	14	26	3	15	9	6	4	1	14
P05023	Sodium/potassium-transporting ATPase subunit alpha-1	ATP1A1	25.6	21	2.949	136	31	12	30	12	11	9	1	7	23
P63000;P60763	Ras-related C3 botulinum toxin substrate 1;Ras-related C3 botulinum toxin substrate 3	RAC1;RAC3	25.5	5	2.461	39	9	2	7	6	3	1	1	2	8
P29966	Myristoylated alanine-rich C-kinase substrate	MARCKS	48.2	8	2.686	76	12	11	8	9	6	10	2	7	11
Q9UBI6	Guanine nucleotide-binding protein G(I)/G(S)/G(O) subunit gamma-12	GNG12	66.7	4	2.523	87	16	9	8	13	9	13	3	4	12
P04899	Guanine nucleotide-binding protein G(i) subunit alpha-2	GNAI2	58.3	15	2.312	281	56	23	48	39	18	27	4	15	51
P62873	Guanine nucleotide-binding protein G(I)/G(S)/G(T) subunit beta-1	GNB1	56.8	14	2.303	139	32	14	21	15	8	14	1	8	26
P35613	Basigin	BSG	44.3	6	2.373	164	39	18	18	15	12	27	1	8	26
P23634	Plasma membrane calcium-transporting ATPase 4	ATP2B4	18.3	17	2.764	119	26	12	9	18	19	16	2	2	15
P62328	Thymosin beta-4;Hematopoietic system regulatory peptide	TMSB4X	47.7	3	1.548	29	3	2	3	3	4	7	1	2	4
P04083	Annexin A1	ANXA1	61.8	18	2.241	261	80	19	23	25	28	26	6	16	38
P09525	Annexin A4;Annexin	ANXA4	41.7	11	3.180	87	23	10	9	13	2	3	2	2	23
P80723	Brain acid soluble protein 1	BASP1	75.3	10	2.798	64	10	7	8	11	2	8	1	4	13
P06733	Alpha-enolase;Enolase	ENO1	32.7	10	2.614	78	17	8	6	10	7	10	1	1	18
Q8WXI7	Mucin-16	MUC16	12.7	49	2.319	773	66	77	130	164	25	67	30	57	157
Q9ULI3	Protein HEG homolog 1	HEG1	10.3	11	2.959	61	9	7	5	15	1	1	0	3	20
P60033	Tetraspanin;CD81 antigen	CD81	35.8	3	2.154	179	26	18	23	24	12	22	6	19	29
P09382	Galectin-1	LGALS1	57.0	6	2.722	82	27	9	7	11	2	2	0	9	15
P13611	Versican core protein	VCAN	5.4	15	2.328	263	76	22	35	30	10	21	13	24	32
P61586;P08134	Transforming protein RhoA;Rho-related GTP-binding protein RhoC	RHOA;RHOC	42.0	7	2.927	88	18	7	14	16	1	8	1	7	16
P60953	Cell division control protein 42 homolog	CDC42	25.1	4	2.664	80	18	11	11	9	5	10	1	4	11
P13987	CD59 glycoprotein	CD59	29.6	4	3.278	86	16	11	10	13	0	14	2	3	17
P04406	Glyceraldehyde-3-phosphate dehydrogenase	GAPDH	36.5	6	1.898	70	16	5	10	9	5	8	2	4	11
P07355;A6NMY6	Annexin A2;Annexin;Putative annexin A2-like protein	ANXA2;ANXA2P2	69.0	23	2.285	557	132	48	68	52	48	72	21	40	76
P62158;P27482	Calmodulin	CALM2;CALM1;CALM3	42.2	8	1.912	123	30	9	15	15	2	17	3	9	23
P08758	Annexin A5;Annexin	ANXA5	70.9	19	3.374	286	106	28	33	31	5	10	6	24	43
P63104	14-3-3 protein zeta/delta	YWHAZ	51.8	12	2.729	163	46	15	16	21	6	17	6	13	23

**Fig 5:**
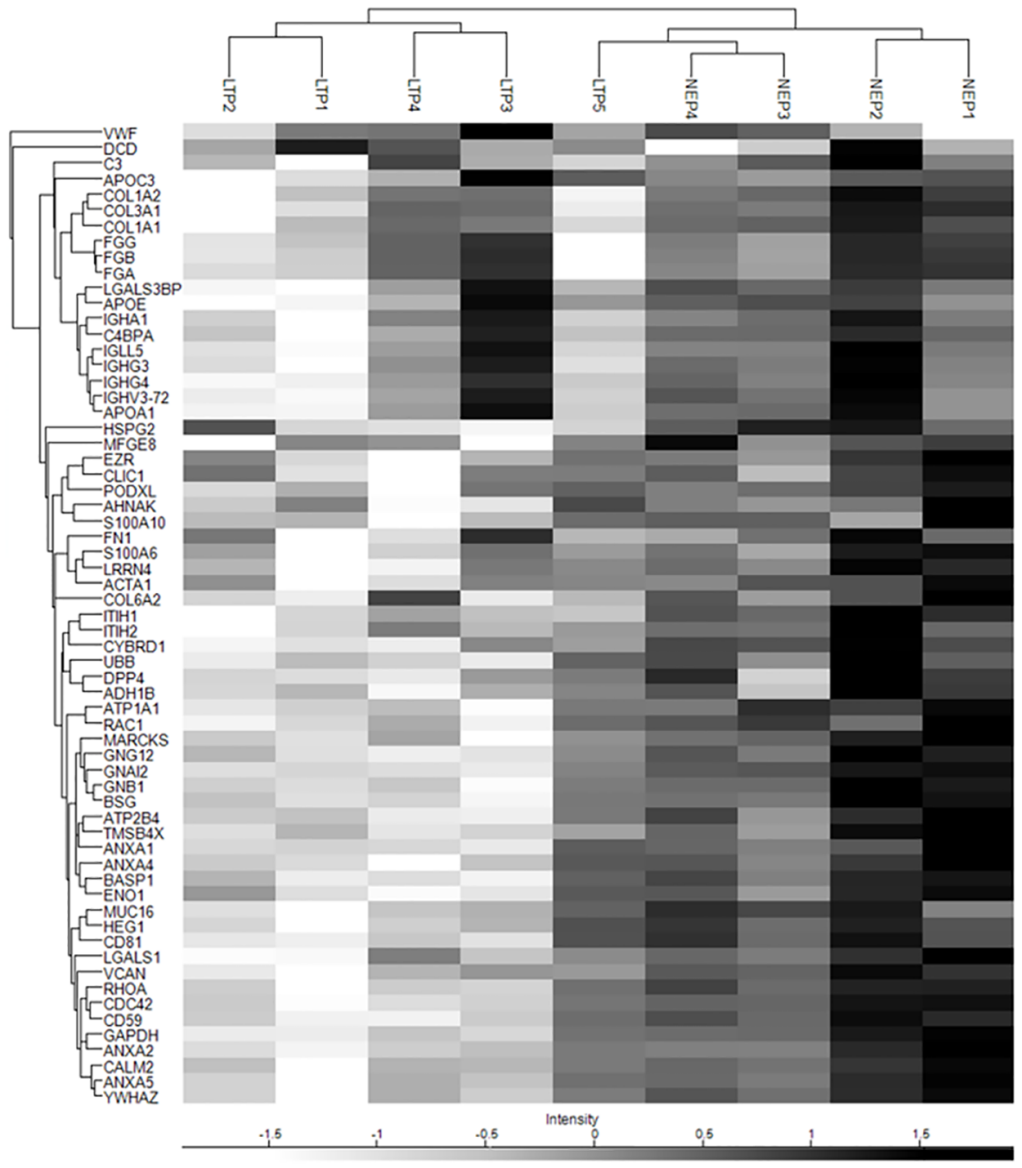
Hierarchical clustering analysis of the 63 “core” proteins. Samples and the 63 proteins shared by all samples were clustered with HCA associated with a heat map. Names of the codifying genes are shown.

**Fig 6:**
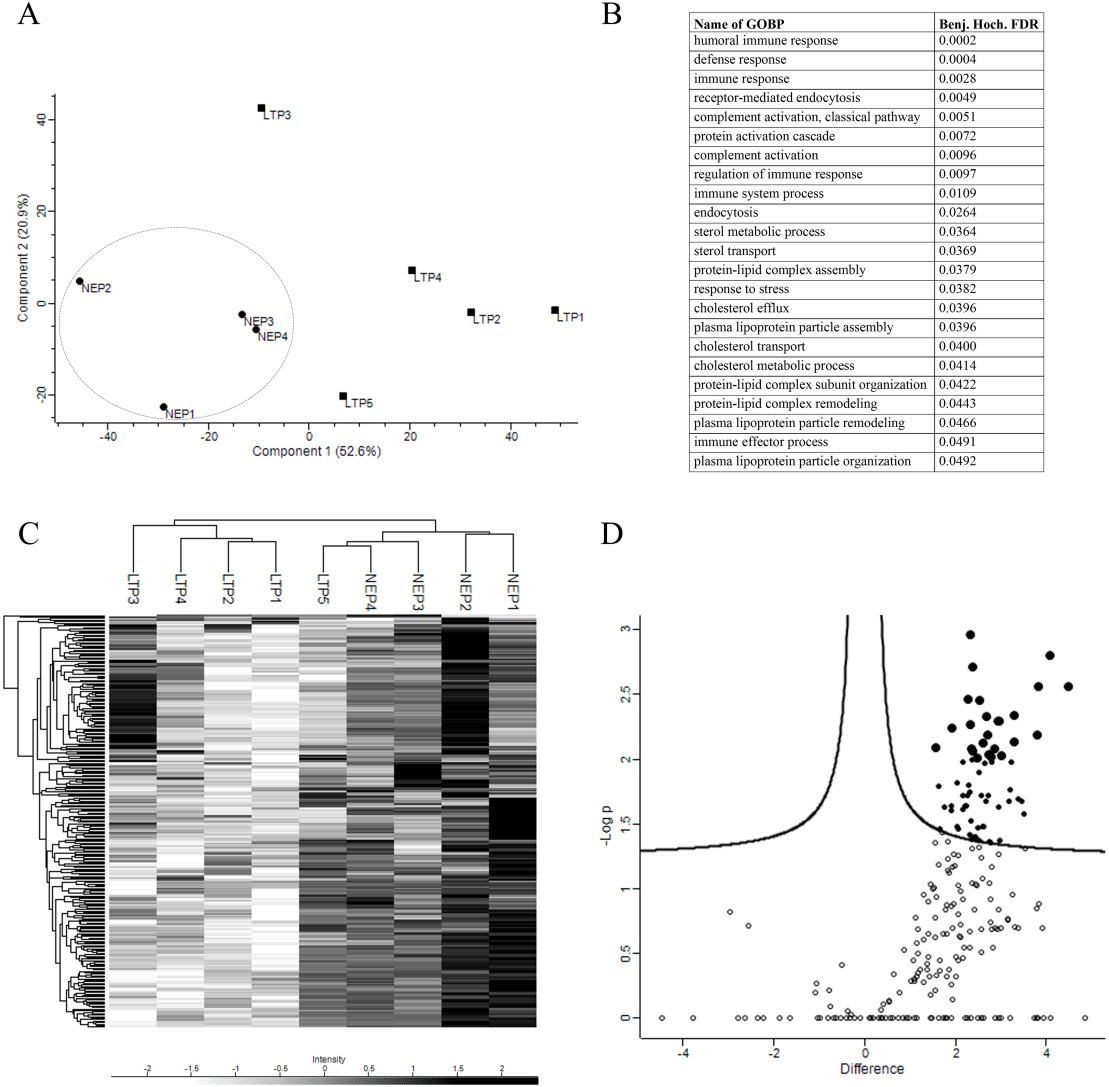
Proteins analyses from PDE-EVs. (A) Two dimensional scatter plot of Principal Component Analysis (PCA) showing component 1 and 2, which account for 52.6% and 20.9%, respectively, the variability of all the 274 proteins. NEPs (circles) and LTPs (squares) are separated by component 1. A dashed line circle indicates grouped NEPs. (B) Table with Gene Ontology biological process enriched terms for component 1 with their corresponding Benjamini-Hochberg FDR values is shown (all the listed terms have a Benj. Hoch. FDR <0.05). (C) HCA associated with a heat map of the 274 proteins (rows) and the samples (columns). (D) A volcano plot was performed to determine significantly differentially expressed proteins between groups. Each circle represents a protein, being statistically significant proteins with this parameters shown as filled circles. Proteins with p-value <0.01 are represented as bigger filled circles.

To further evidence the differences between both groups, a PCA was performed. Based on component 1, which accounts for the 52.6% of the variability between the samples, both groups were segregated based on their time on PD (Fig 6A). The Gene Ontology biological processes analysis of component 1 revealed that most enriched terms in NEP in comparison to LTP are those related to the immune system (Fig 6B). Additionally, HCA of the 274 proteins detected among all samples also clustered most patients based on their time on PD (Fig 6C). Finally, a volcano plot comparing the protein expression between the two groups evidenced the statistically significant proteins showing a significantly different level of expression (Fig 6D). These analyses revealed that up to 67 proteins were significantly overexpressed in NEP than LTP (p-value <0.05).

In addition, a HCA performed exclusively on the 63 "core" proteins identified in all samples resulted in the segregation of 8 out of 9 patients based on time in PD, in a very similar way to the HCA performed with all the proteins (Fig 5).

## Discussion

In this study we report, for the first time to our best knowledge, the presence, isolation and characterization of PDE-EVs from patients on PD.

Peritoneal dialysis is a convenient treatment for end-stage kidney disease patients waiting for a kidney transplant. Studies have reported better survival rates, quality of life and independence of PD patients compared to haemodialysis patients[23,24]. However, during the treatment, fibrotic changes reduce the ultrafiltration capacity of the PM, meaning that many patients have to discontinue treatment.

Current monitoring of the PM function (the PET), requires patients' attendance to the dialysis centre, is time-consuming and only shows alterations when the PM is in an advanced state of fibrosis. Time-delays on identifying PMs' dysfunction may carry dangerous complications, and even lead to the death of the patient. Therefore, finding early biomarkers of PM dysfunction that minimally disturb patients’ daily life may help to overcome these limitations, contribute keeping functional PMs for longer periods and improve patients’ management. Several studies have searched for biomarkers in PDE correlating with PM function, detection of fibrosis, and/or the failure of the technique (reviewed in[25]). Proteomic studies of mesothelial cell lines[7] and transcriptome analysis in rats[26] have reported differences between the protein and miRNA content, respectively, of cells exposed or non-exposed to peritoneal liquids.

It is of current acceptance that information contained in EVs may serve as biomarkers of pathological situations. Biomarkers for kidney pathology have been described in urine EVs[12,27], and serum/plasma EVs have been also related to multiple pathologies[28]. It may be therefore envisaged that PDE-EVs may also provide useful information about the state and function of the PM. Such information could help the clinician to accommodate the treatment to enhance the optimal functionalism of the PM.

PDE-EVs were equally identified in all patients from both NEPs and LTPs groups. SEC-isolated vesicles contained in the tetraspanin rich fractions had a size and morphology compatible with EV, as shown by NTA and cryo-EM analyses. As reported before in urine[29] and plasma samples[30], our results point to SEC as an efficient technique to isolate EVs also from PDE samples. Importantly, SEC permits the segregation of EVs from the bulk of proteins found in samples, thus allowing more accurate analyses of the EV-protein content and enabling the search for minimally expressed proteins. In line, SDS-PAGE results confirmed that EVs were cleanly separated from other major components of the PDE, and preliminary proteomic analysis of SEC-isolated EVs identified a number of well-defined EV-related proteins. Importantly, all these results are in accordance with the recommendations of the International Society for Extracellular Vesicles (ISEV) to identify EVs in a given sample [31], thus validating SEC to isolate EVs also from PDE.

Having identified EVs in all samples, and to further explore possible differences in this pilot study, patients were distributed in two arbitrary groups based on their median time on PD. Both groups did not show major differences in any of the parameters analysed, nor in the PET test. However, a slightly (not significant) reduced number of EVs and also a reduced number of proteins were identified in the PDE-EVs from the LTP group compared to NEPs., It was also interesting to note that a "core" of proteins were identified in both groups, although showing some differences in their level of expression. These "core" proteins included most proteins unequivocally related to EVs. Whether these differences may anticipate a possible worsening of the ultrafiltration capacity of the membrane not detected by PET analyses need further investigation and validation in a wider cohort of patients.

Since this study has consistently demonstrated that EVs can be isolated from concentrated PDE, it seems reasonable to think that these EVs could be used as a source of biomarkers. In addition, the non-invasive origin of the sample and the reduced inconvenience for patients point to the analysis of PDE-EVs as a next step in the definition of early biomarkers of ultrafiltration failure in peritoneal dialysis.

## Supporting information

S1 Table Basal characteristics of the patients. (DOCX)

S2 Table Characteristics of each patients' PET samples analysed. (DOCX)
