# Supplementary material for: Characterization and proteomic profile of extracellular vesicles from peritoneal dialysis efflux
Source: PLoS One. 2017 May 10;12(5):e0176987. doi: 10.1371/journal.pone.0176987 (PMC5425196; doi:10.1371/journal.pone.0176987)
Supplement: S1 Table — (DOCX) [file pone.0176987.s001.docx]

**Supplementary table 1.** Basal characteristics of the patients

| **Patient** | **Age** | **Sex** | **DM** | **HTA** | **Months on PD*** | **CAPD or APD** | **Icodextrin** |
| --- | --- | --- | --- | --- | --- | --- | --- |
| NEP1 | 59 | M | 0 | 1 | 8 | CAPD | 0 |
| NEP2 | 48 | M | 0 | 0 | 10 | CAPD | 1 |
| NEP3 | 62 | M | 0 | 1 | 6 | CAPD | 1 |
| NEP4 | 42 | F | 0 | 1 | 5 | CAPD | 1 |
| LTP1 | 47 | M | 0 | 1 | 21 | CAPD | 1 |
| LTP2 | 54 | F | 0 | 1 | 67 | CAPD | 1 |
| LTP3 | 27 | F | 0 | 1 | 45 | APD | 1 |
| LTP4 | 75 | F | 1 | 1 | 21 | APD | 1 |
| LTP5 | 65 | F | 0 | 0 | 24 | CAPD | 0 |

* Months on peritoneal dialysis at the time of sample collection.

DM, diabetes mellitus; HTA, hypertension; PD, Peritoneal Dialysis; CAPD, Continuous Ambulatory Peritoneal Dialysis; APD, Automated Peritoneal Dialysis; NEP, Newly-Enrolled Patient; LTP, Longer-Treated Patient; 0, absence; 1, presence; F, female; M male.
