# Supplementary material for: Characterization and proteomic profile of extracellular vesicles from peritoneal dialysis efflux
Source: PLoS One. 2017 May 10;12(5):e0176987. doi: 10.1371/journal.pone.0176987 (PMC5425196; doi:10.1371/journal.pone.0176987)
Supplement: S2 Table — (DOCX) [file pone.0176987.s002.docx]

**Supplementary table 2.** Characteristics of each patients' PET samples analysed.

| **Patient** | **PET sample (months on PD)** | **D/P creatinine** | **D/P urea** | **D/D0 glucose** | **UF 240 minutes** | **Transporter** |
| --- | --- | --- | --- | --- | --- | --- |
| NEP1 | 8 | 0.89 | 0.75 | 0.2 | 402 | High |
| NEP2 | 10 | 0.67 | 0.87 | 0.24 | 82 | Medium |
| NEP3 | 6 | 0.83 | 0.85 | 0.2 | 218 | High |
| NEP4 | 5 | 0.64 | 0.82 | 0.26 | 482 | Medium |
| LTP1 | 21 | 0.68 | 0.86 | 0.25 | 254 | Medium |
| LTP2 | 67 | 0.47 | 0.79 | 0.39 | 624 | Low |
| LTP3 | 45 | 0.55 | 0.76 | 0.37 | 676 | Low |
| LTP4 | 21 | 0.75 | 0.89 | 0.24 | 116 | Medium |
| LTP5 | 24 | 0.56 | 0.77 | 0.35 | 577 | Low |

NEP, Newly-Enrolled Patient; LTP, Longer-Treated Patient
